# Practical basket design for binary outcomes with control of family-wise error rate

**DOI:** 10.1186/s12874-023-01872-1

**Published:** 2023-02-27

**Authors:** Junichi Asano, Hiroyuki Sato, Akihiro Hirakawa

**Affiliations:** 1 Biostatistics Group, Center for Product Evaluation, Pharmaceuticals and Medical Devices Agency, Tokyo, Japan; 2 Department of Clinical Biostatistics, Graduate School of Medical and Dental Sciences, Tokyo Medical and Dental University, Tokyo, Japan

**Keywords:** Basket trials, Bayesian approach, Family-wise error rate, Oncology trials, Phase II clinical trials

## Abstract

**Background:**

A basket trial is a type of clinical trial in which eligibility is based on the presence of specific molecular characteristics across subpopulations with different cancer types. The existing basket designs with Bayesian hierarchical models often improve the efficiency of evaluating therapeutic effects; however, these models calibrate the type I error rate based on the results of simulation studies under various selected scenarios. The theoretical control of family-wise error rate (FWER) is important for decision-making regarding drug approval.

**Methods:**

In this study, we propose a new Bayesian two-stage design with one interim analysis for controlling FWER at the target level, along with the formulations of type I and II error rates. Since the difficulty lies in the complexity of the theoretical formulation of the type I error rate, we devised the simulation-based method to approximate the type I error rate.

**Results:**

The proposed design enabled adjustment of the cutoff value to control the FWER at the target value in the final analysis. The simulation studies demonstrated that the proposed design can be used to control the well-approximated FWER below the target value even in situations where the number of enrolled patients differed among subpopulations.

**Conclusions:**

The accrual number of patients is sometimes unable to reach the pre-defined value; therefore, existing basket designs may not ensure defined operating characteristics before beginning the trial. The proposed design that enables adjustment of the cutoff value to control FWER at the target value based on the results in the final analysis would be a better alternative.

## Background

Recent developments in molecular biology and genomics have enabled the classification of patients with common organ-specific cancers into several subpopulations depending on their molecular profiles derived using next-generation genomic sequencing. Clinical development of molecular targeted therapies has recently become pivotal and has been accelerated by the emergence of master protocol trials that assess the combination of several molecular markers and their targeted therapies by employing multiple sub-studies for single or multiple tumor types [1–3]. A basket trial is a type of clinical trial in which eligibility is based on the presence of a specific molecular characteristic across subpopulations with different types of cancers. This feature assumes that a fairly accurate prediction can be made regarding the response of a subpopulation with molecular characteristics to a targeted treatment. Based on this hypothesis, traditional Bayesian hierarchical models (BHMs) based on information borrowing among subpopulations to improve the efficacy of therapeutic effect evaluation are used [4]. However, in some cases, the therapeutic effects among subpopulations with common molecular characteristics are heterogenous, indicating that exchangeability between these subpopulations cannot be assumed. Development of flexible Bayesian methods that account for exchangeable and non-exchangeable subpopulations by extending the BHM or Bayesian model averaging has been garnering interest in addressing the aforementioned issue [5–13]. However, the existing basket designs calibrate the type I and II error rates as well as the family-wise error rate (FWER) based on the results of simulation studies under several selected scenarios because of the methodological nature of Bayesian methods.

Explicit control of the FWER at the target value is also important in basket trials, particularly in registration trials for drug approval [14–16]. In this study, we propose new formulations of subpopulation-specific type I and II error rates and FWER by extending the type I error rate in Bayesian sequential design for single-arm phase II trials with binary outcomes proposed by Shi and Yin (2019) [17]. Based on these formulations, we developed a two-stage Bayesian design with a binary endpoint controlling the FWER. In the first stage, we performed futility analysis to exclude subpopulations without efficacy based on the Bayesian posterior probability. In the second stage, we determined whether targeted therapy was effective for each subpopulation. The proposed design enables adjustment of the cutoff value to control the FWER in the final analysis according to the number of accumulated patients. Using the proposed method, the exact type I and II error rates and FWER can be calculated; however, it involves time-consuming numerical experiments to determine the sample size. We, therefore, devise a time-saving simulation-based method to approximate the type I error rate. Simulation studies were conducted under various scenarios and settings to evaluate the operating characteristics of the proposed method.

The proposed design is presented in the Methods section. The operating characteristics of the proposed design are described in the Results sections. Finally, the utility of the proposed design is summarized in the Discussion and conclusion section.

## Methods

This section is divided into four parts: the trial framework, the estimation method for posterior probability, the definitions of type I/II error rates and FWER, and the numerical calculation method for type I/II error rates.

### Trial framework

We propose a two-stage design using binary endpoints with *J* ($j = 1, \cdots , J$) subpopulations. The notation of the trial framework is presented in Table 1. For each subpopulation, the trial performs one interim analysis for futility stopping when the number of patients included in that subpopulation reaches a pre-specified value. Consequently, *J* interim analyses were performed. Subpopulation *j* was excluded from the study when the futility stopping boundary was obtained. The final analysis was performed when all remaining subpopulations achieved a pre-defined number of subjects. Supposing that $\{-j\}$ refers to the index of the subpopulations excluding subpopulation *j*, $\{-j\}$ can be calculated as $\{-j\} \ (=\{1,\cdots ,j-1,j+1,\cdots ,J\})$.

**Table 1:** Notation of trial framework

$n_j^{*}$: Number of patients for subpopulation *j* during interim analysis
$n_{\{-j\}}^{*}$: Number of patients for subpopulation $\{-j\}$ during interim analysis for subpopulation *j*
$r_j^{*}$: Number of responses for subpopulation *j* during interim analysis
$r_{\{-j\}}^{*}$: Number of responses for subpopulation $\{-j\}$ during interim analysis for subpopulation *j*
$n_j$: Number of patients for subpopulation *j* during final analysis
$n_{\{-j\}}$: Number of patients for subpopulation $\{-j\}$ during final analysis
$r_j$: Number of responses for subpopulation *j* during final analysis
$r_{\{-j\}}$: Number of responses for subpopulation $\{-j\}$ during final analysis
$p_j$: True response rate for subpopulation *j*
$p_{\{-j\}}$: True response rate for subpopulation $\{-j\}$
$p_0$: Response rate to the standard treatment (i.e., null response rate), which was set as a constant among subpopulations
$p_1$: Alternative expected response rate which, was set as a constant among subpopulations

In the interim analysis for subpopulation *j*, we estimated the posterior probability $\Pr (p_j >p_0 \mid \textbf{n}^{*},\textbf{r}^{*})$, where $\textbf{n}^{*}$ and $\textbf{r}^{*}$ represent the vectors of data with respect to the number of patients and responses in interim analysis for subpopulation *j*, respectively. Subpopulation *j* was excluded from the study when the posterior probability of $\Pr (p_j>p_0 \mid \textbf{n}^{*},\textbf{r}^{*})$ was less than the prespecified threshold of $c_{1,j}$.

1 $$\begin{aligned} \Pr (p_j >p_0 \mid \textbf{n}^{*},\textbf{r}^{*} ) < c_{1,j} \ . \end{aligned}$$

In final analysis after completing patient enrollment, we estimated $\Pr (p_j>p_0 \mid \textbf{n},\textbf{r})$ where $\textbf{n}$ and $\textbf{r}$ refer to the vectors of data with respect to the number of patients and responses in the final analysis, respectively. Notably, $n_j=n_j^{*}$ at the final analysis when the subpopulation *j* satisfied the futility criterion during the interim analysis for subpopulation *j*. The investigational treatment was declared as effective in subpopulation *j* when the posterior probability of $\Pr (p_j>p_0 \mid \textbf{n},\textbf{r})$ exceeded the pre-specified threshold of $c_{2,j}$.

2 $$\begin{aligned} \Pr (p_j>p_0 \mid \textbf{n},\textbf{r} ) > c_{2,j} \ . \end{aligned}$$

The two thresholds, $c_{1,j}$ and $c_{2,j}$, were determined for each subpopulation to maintain FWER at the target level using the method described in the Numerical calculation section.

### Posterior probability estimation

We estimate the posterior probabilities of $\Pr (p_j >p_0 \mid \textbf{n}^{*},\textbf{r}^{*} )$ of Eq. (1) and $\Pr (p_j>p_0 \mid \textbf{n},\textbf{r})$ of Eq. (2) based on the BHM [18]. $\theta _j$ itself be a log-odds $\theta _j=\textrm{logit} (p_j)$; the prior probability of $\theta _j$ sets the normal distribution with mean $\mu$ and variance $\tau ^{-2}$ as follows:

3 $$\begin{aligned} \theta _j \mid \mu , \tau \sim Normal(\mu ,\tau ^{-2}) \ . \end{aligned}$$

The hyperprior parameter of $\mu$ is assumed to follow a normal distribution with a mean $\tilde{\mu }$ and variance $\tilde{\sigma }^2$,

4 $$\begin{aligned} \mu \sim Normal(\tilde{\mu },\tilde{\sigma }^2) \ . \end{aligned}$$

The hyperprior parameter of $\tau$ is assumed to follow the gamma distribution with mean $\nu /\xi$ and variance $\nu /\xi ^2$.

5 $$\begin{aligned} \tau \sim Gamma(\nu ,\xi ) \ . \end{aligned}$$

The posterior joint distribution for the parameters for subpopulation *j* in the final analysis (replace $\textbf{n}$ with $\textbf{n}_j^{*}$ for interim analysis) is as follows:

6 $$\begin{aligned} f( \varvec{\theta } ,\mu ,\tau \mid \textbf{n},\textbf{r}) = \left\{ \prod _{j=1}^{J} L(\theta _j \mid n_j, r_j) f(\theta _j \mid \mu , \tau )\right\} f(\mu \mid \tilde{\mu }, \tilde{\sigma }^2) f(\tau \mid \nu , \xi ) \ , \end{aligned}$$

where $$L(\theta _j \mid n_j, r_j) = \left( {\begin{array}{c}n_j\\ r_j\end{array}}\right) p_j^{r_j}(1-p_j)^{n_j-r_j}$$,and $\varvec{\theta }=(\theta _1,\theta _2,\cdots , \theta _J)$ is the vector of the log odds for the true response rate for subpopulation *j*, and *f* is the probability density function for each parameter.

In the proposed method, $\Pr (p_j>p_0 \mid \textbf{n},\textbf{r})$ of Eq. (2) is calculated considering the prior distribution of $p_0$ based on Thall and Simon (1994) [18] as follows:

7 $$\begin{aligned} \Pr (p_j>p_0 \mid \textbf{n},\textbf{r}) = \int _{0}^{1} \left\{ 1-F(p_0 \mid \textbf{n},\textbf{r}) \right\} Beta(p_0 ; a_0, b_0)dp_0\ , \end{aligned}$$

where $p_0$ is assumed to follow the beta distribution with hyperparameters $a_0$ and $b_0$. The values of $a_0$ and $b_0$ are set as the mean of the beta distribution on $p_0$ (i.e., $p_0=a_0/(a_0+b_0)$) based on historical information. $F(p_j \mid \textbf{n},\textbf{r})$ are the cumulative distribution functions of $p_j$ calculated from the posterior samples of the posterior joint distribution of Eq. (6) using the Hamilton Monte Carlo method.

### Definitions

#### Subpopulation-specific type I error rate and FWER

The type I error rate in the Bayesian sequential design with the binary endpoint proposed by Shi and Yin (2019) [17] was defined as the sum of the probabilities for all possible cases in which the truly ineffective treatment was incorrectly declared as effective. As the response rates in the basket trials can be correlated among subpopulations, and the BHM borrows information among subpopulations, formulating the definition of subpopulation-specific type I error rate should account for the observed response rates of the remaining subpopulations. To this end, we first introduce the probability of declaring the therapeutic effect in the final analysis under a null response rate of $p_0$ for the subpopulation *j*.

8 $$\begin{aligned} \tilde{\alpha _j}(\textbf{n}^{*},\textbf{r}^{*},\textbf{n},\textbf{r})= & {} \sum _{r_j^{*}=0}^{n_j^{*}}\sum _{r_j=r_j^{*}}^{n_j-n_j^{*}+r_j^{*}} \left\{ I\left[ \Pr (p_j>p_0 \mid \textbf{n}^{*},\textbf{r}^{*} ) \ge c_{1,j} \right] Bin(r_j^{*};n_j^{*},p_0) \right. \nonumber \\{} & {} \qquad \left. I\left[ \Pr (p_j>p_0 \mid \textbf{n},\textbf{r} ) > c_{2,j} \right] Bin(r_j-r_j^{*};n_j-n_j^{*},p_0) \right\} \ , \end{aligned}$$

where *I* indicates the indicator function and *Bin*() is the probability density function of the binomial distribution. We extend $\tilde{\alpha _j}(\textbf{n}^{*},\textbf{r}^{*},\textbf{n},\textbf{r})$ to the subpopulation-specific type I error rate for subpopulation *j*, which accounts for all possible combinations of $n_{\{-j\}}^{*}$,$r_{\{-j\}}^{*}$,$n_{\{-j\}}$, $r_{\{-j\}}$ as follows:

9 $$\begin{aligned} \alpha _j = \frac{\sum _{n_{j^{\prime }\in \{-j\}}^{*}=0}^{n_{j^{\prime }}} \sum _{r_{j^{\prime }\in \{-j\}}^{*}=0}^{n_{j^{\prime }}^{*}}\sum _{r_{j^{\prime }\in \{-j\}}=r_{j^{\prime }}^{*}}^{n_{j^{\prime }}-n_{j^{\prime }}^{*}+r_{j^{\prime }}^{*} } \left[ w\tilde{\alpha _j}(\textbf{n}^{*},\textbf{r}^{*},\textbf{n},\textbf{r})\right] }{\sum _{n_{j^{\prime }\in \{-j\}}^{*}=0}^{n_{j^{\prime }}} \sum _{r_{j^{\prime }\in \{-j\}}^{*}=0}^{n_{j^{\prime }}^{*}}\sum _{r_{j^{\prime }\in \{-j\}}=r_{j^{\prime }}^{*}}^{n_{j^{\prime }}-n_{j^{\prime }}^{*}+r_{j^{\prime }}^{*} } w} \ , \end{aligned}$$

where $w=\prod _{j^{\prime }\in \{-j\}}Bin(r_{j^{\prime }}^{*};n_{j^{\prime }}^{*},p_{j^{\prime }})Bin(r_{j^{\prime }}-r_{j^{\prime }}^{*};n_{j^{\prime }}-n_{j^{\prime }}^{*},p_{j^{\prime }})$; i.e., $\alpha _j$ is the weighted average of $\tilde{\alpha _j}(\textbf{n}^{*},\textbf{r}^{*},\textbf{n},\textbf{r})$ for all possible values with respect to $n_{\{-j\}}^{*}$,$r_{\{-j\}}^{*}$,$n_{\{-j\}}$, and $r_{\{-j\}}$ for the subpopulations $\{-j\} \ (=\left\{ 1,\cdots ,j-1,j+1,\cdots ,J \right\} )$, which are weighted by the corresponding probability of *w*. As possible values, we also consider the values of pattern with which the subpopulation *j* met the futility criterion at the interim analysis for subpopulation *j*, and then we set $n_j=n_j^{*}$ with that pattern. The FWER is defined as $\alpha _{FWER}=1-\prod _{j=1}^J \left\{ 1-\alpha _j \right\}$ under the assumption that the true response rates in all subpopulations represent null response rates, $p_j=p_0$ ($j=1,\cdots ,J$).

#### Subpopulation-specific type II error rate

To define the subpopulation-specific type II error rate for subpopulation *j*, we also introduced the probability of declaring the therapeutic effect during the final analysis with the alternative response rate of $p_1$ for subpopulation *j* as follows:

10 $$\begin{aligned} \tilde{\beta _j}(\textbf{n}^{*},\textbf{r}^{*},\textbf{n},\textbf{r})=\sum _{r_j^{*}=0}^{n_j^{*}}\sum _{r_j=r_j^{*}}^{n_j-n_j^{*}+r_j^{*}} \left\{ I\left[ \Pr (p_j>p_0 \mid \textbf{n}^{*},\textbf{r}^{*} ) < c_{1,j} \right] Bin(r_{j}^{*};n_j^{*},p_1) \nonumber \right. \\ \left. + I\left[ \Pr (p_j>p_0 \mid \textbf{n}^{*},\textbf{r}^{*} ) \ge c_{1,j} \right] Bin(r_j^{*};n_j^{*},p_1) \right. \nonumber \\ \left. I\left[ \Pr (p_j >p_0 \mid \textbf{n},\textbf{r} ) \le c_{2,j} \right] Bin(r_j-r_j^{*};n_j-n_j^{*},p_1) \right\} \ , \end{aligned}$$

to obtain the subpopulation-specific type II error rate for subpopulation *j*.

11 $$\begin{aligned} \beta _{j} = \frac{\sum _{n_{j^{\prime }\in \{-j\}}^{*}=0}^{n_{j^{\prime }}} \sum _{r_{j^{\prime }\in \{-j\}}^{*}=0}^{n_{j^{\prime }}^{*}}\sum _{r_{j^{\prime }\in \{-j\}}=r_{j^{\prime }}^{*}}^{n_{j^{\prime }}-n_{j^{\prime }}^{*}+r_{j^{\prime }}^{*} } \left[ v\tilde{\beta _j}(\textbf{n}^{*},\textbf{r}^{*},\textbf{n},\textbf{r})\right] }{\sum _{n_{j^{\prime }\in \{-j\}}^{*}=0}^{n_{j^{\prime }}} \sum _{r_{j^{\prime }\in \{-j\}}^{*}=0}^{n_{j^{\prime }}^{*}}\sum _{r_{j^{\prime }\in \{-j\}}=r_{j^{\prime }}^{*}}^{n_{j^{\prime }}-n_{j^{\prime }}^{*}+r_{j^{\prime }}^{*} } v} \ , \end{aligned}$$

where $v=\prod _{j^{\prime }\in \{-j\}}Bin(r_{j^{\prime }}^{*};n_{j^{\prime }}^{*},p_{j^{\prime }})Bin(r_{j^{\prime }}-r_{j^{\prime }}^{*};n_{j^{\prime }}-n_{j^{\prime }}^{*},p_{j^{\prime }})$. We define the subpopulation-specific power for the subpopulation *j* as $1-\beta _j$.

### Numerical calculation

Extensive calculations are required to determine the values of $\alpha _j$, $\beta _j$, and $\alpha _{FWER}$, even when the number of subpopulations is small. For example, when calculating $\alpha _j$ with $J=4$, $n_j^{*}=10$ ($j=1,\cdots ,J$) and $n_j=20$ ($j=1,\cdots ,J$), the calculations are repeated for 700 million combinations of the values of $\textbf{r}^{*}$ and $\textbf{r}$; therefore, the burdens and time required to perform calculations for obtaining the exact values of of $\alpha _j$, $\beta _j$, and $\alpha _{FWER}$ are enormous and unrealistic. Instead of the exact values of $\alpha _j$, $\beta _j$, and $\alpha _{FWER}$, we propose to use values of $\alpha _j$, $\beta _j$, and $\alpha _{FWER}$ calculated using simulated data and call those the well-approximated $\alpha _j$, $\beta _j$, and $\alpha _{FWER}$ in this study. To this end, we devised a numerical approach for determining the thresholds that can approximately control the FWER at the target level. Specifically, given the values of $n_j^{*}$, $n_j$, $p_0$, $p_1$, $a_0$, $b_0$, $c_{1,j}$ and $c_{2,j}$ ($j=1,\cdots ,J$), we calculated the well-approximated values of $\alpha _j$ and $\beta _j$ using simulated data in five steps as described in the following paragragh. Subsequently, well-approximated FWER was calculated from $\alpha _{FWER}=1-\prod _{j=1}^J \left\{ 1-\alpha _j \right\}$ using the well-approximated values of $\alpha _j$.

Step 1 We set the parameters of $n_j^{*}$, $n_j$, $p_0$, $p_1$, $a_0$, $b_0$ ($j=1,\cdots ,J$), under which the arbitrary ranges of $c_{1,j}$ and $c_{2,j}$ were specified, and all combinations of these values were enumerated.
Step 2 We randomly selected subpopulation *j* from a multinomial distribution with equal success probability among subpopulations and generated a binary response data for a patient from the Bernoulli distribution with the true response rate (i.e., $p_0$ or $p_1$) in the subpopulation *j*. This step was repeated until the number of patients in any subpopulation reached $n_j^{*}$ ($j=1,\cdots ,J$).
Step 3 After reaching $n_j^{*}$ patient enrollment in subpopulation *j*, futility evaluation based on Eq. (1) is performed. If subpopulation *j* satisfied the futility criterion in the interim analysis, subpopulation *j* was excluded from the trial. Otherwise, patient enrollment was continued for subpopulation *j*.
Step 4 After completing patient enrollment for all subpopulations except for the excluded subpopulations in the previous step, the posterior probability for subpopulation *j* was evaluated based on Eq. (2).
Step 5 Steps 2–4 were repeated up to 10,000 times, and the empirical values of $\alpha _j$ (i.e., proportion of declaration that the investigational treatment was effective under the assumption of $p_0$) and $\beta _j$ (i.e., proportion of declaration that the investigational treatment was ineffective under the assumption of $p_1$) were calculated.

Throughout these steps, determination of the two thresholds, $c_{1,j}$ and $c_{2,j}$, was explored for each subpopulation to maintain a well-approximated FWER of $\alpha _{FWER}$ at the target level. If two and more combinations of $c_{1,j}$ and $c_{2,j}$ were obtained to control the well-approximated FWER $< 5\%$, then we selected the values of $c_{1,j}$ and $c_{2,j}$ with the lowest average of well-approximated values of $\beta _j$ among subpopulations with the true response rates in all subpopulations are under $p_1$, i.e., $p_j = p_1 (j=1,\cdots ,J)$.

## Results

We evaluated the operating characteristics through simulation studies. This section is divided into four parts: simulation settings, investigation of the accuracy of the numerical approach, simulation results under various scenarios, and operating characteristics with different sample sizes among subpopulations.

### Simulation settings

We evaluated the operating characteristics of the proposed method in a simulation study. The values of $p_0$ and $p_1$ were set to 0.05 and 0.25, respectively. The number of subpopulations *J* was set to four. The true response rates of each subpopulation assumed in each scenario with $J=4$ are listed in Table 2. The maximum number of accrual patients in each subpopulation was 20 (i.e., $n_j=20$). For each subpopulation, one interim analysis for futility was conducted when the number of accrued patients reached 10 (i.e., $n_j^{*}=10$). We assumed that $p_0$ followed a beta distribution with parameters *Beta*(10, 190) and a mean of 0.05 based on the analysis reported by Hirakawa et al. (2018) [19].

**Table 2:** True response rates for four subpopulations ($J=4$)

Scenario *k*	Subpopulation *j*			
	1	2	3	4
1	0.05	0.05	0.05	0.05
2	0.05	0.05	0.05	0.25
3	0.05	0.05	0.25	0.25
4	0.05	0.25	0.25	0.25
5	0.25	0.25	0.25	0.25

In the proposed method, we used $\tilde{\mu } = -1.734$ (i.e., $p_j=(0.05+0.25)/2=0.15$) and $\tilde{\sigma }^2=10$ with a weak (or strong) degree of borrowing information, i.e., ($\nu ,\xi$)=(2, 20) (or (2, 2)). We estimated the posterior distribution using the rstan package in R software.

In simulation studies, we compared the proposed method with an independent method. For the independent method, the posterior distributions of $\Pr ( p_j > p_0 \mid \textbf{n}^{*},\textbf{r}^{*})$ and $\Pr ( p_j > p_0 \mid \textbf{n},\textbf{r} )$ were calculated independently as follows:

12 $$\begin{aligned} \Pr (p_j>p_0 \mid \textbf{n}^{*},\textbf{r}^{*}) = \int _{0}^{1} \left\{ 1-F_{Beta}(p_0 \mid a_{ind}+r_j^{*}, b_{ind}+n_j^{*}-r_j^{*}) \right\} \nonumber \\ Beta(p_0 ; a_0, b_0)dp_0\ , \end{aligned}$$

13 $$\begin{aligned} \Pr (p_j>p_0 \mid \textbf{n},\textbf{r}) = \int _{0}^{1} \left\{ 1-F_{Beta}(p_0 \mid a_{ind}+r_j, b_{ind}+n_j-r_j) \right\} \nonumber \\ Beta(p_0 ; a_0, b_0)dp_0\ , \end{aligned}$$

where $F_{Beta}$ refered the distribution function of the beta distribution, and $a_{ind}$ and $b_{ind}$ were set to 0.6 and 1.4, respectively, based on the analysis reported by Hirakawa et al. (2018) [19].

We conducted 10,000 simulations for each scenario. We have presented well-approximated type I error rates, power (i.e., one minus type II error rate), and FWER here.

### Accuracy of numerical approach for determining thresholds for declaration of the therapeutic effect

Before conducting the simulation studies, we examined the accuracy of the numerical values for $c_1$ and $c_2$ determined using the numerical approach described in the Numerical calculation section. We used common values for $c_{1,j}$ and $c_{2,j}$ between subpopulations (i.e., $c_1 = c_{1,1} = \cdots = c_{1,J}$ and $c_2 = c_{2,1}=\cdots =c_{2,J}$) because the true null and alternative response rates were the same among subpopulations. To reduce the computational burden of the calculations, we compared the exact FWER with a well-approximated value using the proposed numerical approach in the independent method. We set the ranges for $c_{1}$ and $c_{2}$ from 0.2 to 0.4 in increments of 0.1 and from 0.95 to 0.99 in increments of 0.01. Other simulation settings were the same as those described in the Simulation settings section. The exact FWER was calculated from $1-\prod _{j=1}^{J} \left\{ 1-\tilde{\alpha _j} (n_j^{*},r_j^{*},n_j,r_j) \right\}$ because the values of the proposed numerical method in the independent method were not affected by the results of other subpopulations. Table 3 shows the exact and well-approximated FWER under Scenario 1. We found that the observed differences between the exact and well-approximated values of FWER were negligible.

**Table 3:** Exact and well-approximated values of family-wise error rate (FWER)

$c_1$	$c_2$	Exact value	Well-approximated value
0.2	0.95	26.9%	26.2%
	0.96	6.2%	6.7%
	0.97	6.2%	6.7%
	0.98	6.2%	6.7%
	0.99	1.0%	0.6%
0.3	0.95	26.9%	26.2%
	0.96	6.2%	6.7%
	0.97	6.2%	6.7%
	0.98	6.2%	6.7%
	0.99	1.0%	0.6%
0.4	0.95	24.7%	24.5%
	0.96	6.0%	5.9%
	0.97	6.0%	5.9%
	0.98	6.0%	5.9%
	0.99	1.0%	0.9%

### Simulation results

For the hyperprior parameter of $\tau$, which is assumed to be followed by the gamma distribution with mean $\nu /\xi$ and variance $\nu /\xi ^2$, $(\nu ,\xi )=(2,20)$ and (2, 2) were used in the proposed method with weak and strong information borrowing, respectively. In the following simulation studies, the values of $c_1$ and $c_2$ were selected to achieve a well-approximated FWER of lower than and nearest to 5% using the proposed numerical approach. The ranges for $c_{1}$ and $c_{2}$ were 0.2–0.4 in increments of 0.1 and of 0.95–0.99 in increments of 0.01. We used $c_1=0.2$ and $c_2=0.98$ for the proposed method with weak information borrowing, $c_1=0.2$ and $c_2=0.95$ for the proposed method with strong information borrowing, and $c_1=0.2$ and $c_2=0.99$ for the independent method. Using these values for $c_1$ and $c_2$, the well-approximated FWER for independent, BHM-weak, and BHM-strong methods were 1%, 4%, and 4%, respectively. Note that the empirical FWER (i.e., proportion of declaration that investigational treatment was effective in at least one subpopulation among all subpopulations under the assumption that true response rates in all subpopulations are $p_0$ (i.e., scenario 1)) for independent, BHM-weak, and BHM-strong methods were 1%, 4%, and 4%, respectively . We found that the observed differences between the well-approximated and empirical values of FWER were negligible.

Table 4 shows the well-approximated type I error rates and power when using $c_1$ and $c_2$ which were selected to maintain a well-approximated FWER $<5\%$ in 10,000 simulations. In the four scenarios in which at least one subpopulation had a true response rate of 0.05 (in scenarios 1, 2, 3, and 4), the average well-approximated type I error rates of independent, BHM-weak, and BHM-strong methods across the four subpopulations were 0%, 1%, and 3%, respectively.

**Table 4:** Well-approximated type I error rates and power observed when controlling the well-approximated FWER below 5%

		Subpopulation *j*			
Scenario	Method	1	2	3	4
1	Independent	**0%**	**0%**	**0%**	**0%**
	BHM-weak	**1%**	**1%**	**1%**	**1%**
	BHM-strong	**1%**	**1%**	**1%**	**1%**
2	Independent	**0%**	**0%**	**0%**	54%
	BHM-weak	**1%**	**1%**	**2%**	67%
	BHM-strong	**2%**	**3%**	**4%**	65%
3	Independent	**0%**	**0%**	61%	53%
	BHM-weak	**1%**	**1%**	73%	67%
	BHM-strong	**5%**	**7%**	83%	76%
4	Independent	**0%**	57%	61%	53%
	BHM-weak	**1%**	71%	74%	68%
	BHM-strong	**10%**	85%	91%	85%
5	Independent	57%	57%	61%	53%
	BHM-weak	72%	71%	75%	68%
	BHM-strong	91%	90%	95%	90%

*BHM* Bayesian hierarchical models

Type I error rates are shown in boldface; values not presented in bold indicate power

Under the four scenarios in which at least one subpopulation had a true response rate of 0.25 (i.e., scenarios 2, 3, 4, and 5), the average well-approximated power of independent, BHM-weak, and BHM-strong methods across subpopulations were 57%, 71%, and 85%, respectively. The well-approximated power of the BHM-strong methods increased with the increase in the number of subpopulations, with a true response rate of 0.25. In the scenario with all subpopulations showing a true response rate of 0.25 (i.e., scenario 5), the averaged well-approximated power of independent, BHM-weak, and BHM-strong methods across subpopulations were 57%, 71%, and 91%, respectively.

### Operating characteristics with different samples sizes among subpopulations

We often encounter a situation in which the number of enrolled patients differs from that determined at the planning stage of the trials. In this section, the operating characteristics of the proposed method were calculated using the sample sizes at the final analysis $({\mathrm n}_1,{\mathrm n}_2,{\mathrm n}_3,{\mathrm n}_4)=(20,20,20,5),(20,20,20,1),(20,20,20,30),(5,5,5,40),\;\mathrm{and}\;(15,\;10,\;5,\;1)$ in scenario 1 with the values of $c_1$ and $c_2$. These values were selected to achieve a well-approximated FWER of lower than and closest to 5% with the sample size of $(n_1, n_2, n_3, n_4) = (20, 20, 20, 20)$ at the planning stage of the trials. Other simulation settings were the same as those used in the previous section.

Table 5 shows well-approximated FWER under the scenario 1 using different sample sizes among subpopulations. According to Table 5, the BHM-strong method controlled the well-approximated FWER to below 5%. The well-approximated FWER exceeded the target level 5% when using $(n_1, n_2, n_3, n_4) = (5, 5, 5, 40)$ for the independent method and $(n_1, n_2, n_3, n_4) = (20, 20, 20, 1)$ and (15, 10, 5, 1) for the BHM-weak method. To suppress this inflation of FWER, the value of $c_2$ was re-determined using the actual sample size at final analysis. For the independent method, $c_2$ was re-determined as 0.995, and the well-approximated FWER was $0.4\%$ under $(n_1, n_2, n_3, n_4) = (5, 5, 5, 40)$. For the BHM-weak method, $c_2$ was re-determined as 0.99, and the well-approximated FWER were 4.3% and 4.8% with $(n_1, n_2, n_3, n_4) = (20, 20, 20, 1)$ and $(n_1, n_2, n_3, n_4) = (15, 10, 5, 1)$, respectively.

**Table 5:** Well-approximated FWER under scenario 1 using different sample sizes among subpopulations

	FWER		
Sample size of ($n_1$, $n_2$, $n_3$, $n_4$)	Independent	BHM-weak	BHM-strong
(20, 20, 20, 5)	3.1%	3.8%	3.1%
(20, 20, 20, 1)	0.4%	7.1%	3.4%
(20, 20, 20, 30)	0.7%	3.2%	3.4%
(5, 5, 5, 40)	6.1%	4.6%	3.4%
(15, 10, 5, 1)	3.3%	7.9%	3.4%

*BHM* Bayesian hierarchical models

### Operating characteristics with weak informative prior for the variance $\tilde{\sigma }^2$ of BHM

We investigated the operating characteristics of the proposed method with BHM under the assumption of weak informative prior for the variance $\tilde{\sigma }^2$ of the hyperprior parameter of $\mu$ (Table 6). We set $\tilde{\sigma }^2$ to 25 or 50; other simulation settings were the same as those described in the Simulation settings section. In the following simulation studies, the values of $c_1$ and $c_2$ were selected to achieve a well-approximated FWER of lower than and nearest to 5% using the proposed numerical approach.

**Table 6:** Well-approximated type I error rates and power with weak informative prior for variance of BHM

			Subpopulation *j*			
Scenario	Method	$\tilde{\sigma }^2$	1	2	3	4
1	BHM-weak	$\tilde{\sigma }^2 = 25$	**1%**	**1%**	**1%**	**1%**
		$\tilde{\sigma }^2 = 50$	**1%**	**1%**	**1%**	**1%**
	BHM-strong	$\tilde{\sigma }^2 = 25$	**1%**	**1%**	**2%**	**1%**
		$\tilde{\sigma }^2 = 50$	**1%**	**1%**	**2%**	**1%**
2	BHM-weak	$\tilde{\sigma }^2 = 25$	**1%**	**1%**	**1%**	66%
		$\tilde{\sigma }^2 = 50$	**0%**	**1%**	**1%**	66%
	BHM-strong	$\tilde{\sigma }^2 = 25$	**2%**	**4%**	**5%**	70%
		$\tilde{\sigma }^2 = 50$	**2%**	**3%**	**4%**	71%
3	BHM-weak	$\tilde{\sigma }^2 = 25$	**1%**	**1%**	73%	67%
		$\tilde{\sigma }^2 = 50$	**1%**	**1%**	72%	67%
	BHM-strong	$\tilde{\sigma }^2 = 25$	**6%**	**8%**	85%	78%
		$\tilde{\sigma }^2 = 50$	**6%**	**8%**	85%	78%
4	BHM-weak	$\tilde{\sigma }^2 = 25$	**1%**	71%	74%	68%
		$\tilde{\sigma }^2 = 50$	**1%**	71%	74%	67%
	BHM-strong	$\tilde{\sigma }^2 = 25$	**15%**	87%	93%	87%
		$\tilde{\sigma }^2 = 50$	**15%**	87%	92%	87%
5	BHM-weak	$\tilde{\sigma }^2 = 25$	72%	71%	75%	69%
		$\tilde{\sigma }^2 = 50$	72%	71%	74%	68%
	BHM-strong	$\tilde{\sigma }^2 = 25$	93%	93%	96%	92%
		$\tilde{\sigma }^2 = 50$	93%	92%	96%	92%

*BHM* Bayesian hierarchical models

Type I error rates are shown in boldface; values not presented in bold indicate power

Table 6 shows the well-approximated type I error rates and power in scenarios 1-5. The values of well-approximated type I error rates and power were very similar with assumptions of not only $\tilde{\sigma }^2 = 25$ and 50, but also $\tilde{\sigma }^2 = 10$ in Table 4. Therefore, we found that the value of the variance $\tilde{\sigma }^2$ of the hyperprior parameter of $\mu$ did not affect the operating characteristics of the proposed method.

**Table 7:** Well-approximated type I error rates and power observed with weak informative prior distribution for $p_0$

		Subpopulation *j*			
Scenario	Method	1	2	3	4
1	Independent	**0%**	**0%**	**0%**	**0%**
	BHM-weak	**0%**	**0%**	**0%**	**0%**
	BHM-strong	**0%**	**0%**	**0%**	**0%**
2	Independent	**0%**	**0%**	**0%**	54%
	BHM-weak	**0%**	**0%**	**0%**	54%
	BHM-strong	**0%**	**1%**	**1%**	54%
3	Independent	**0%**	**0%**	61%	54%
	BHM-weak	**0%**	**0%**	61%	54%
	BHM-strong	**1%**	**2%**	68%	62%
4	Independent	**0%**	57%	61%	53%
	BHM-weak	**0%**	57%	61%	53%
	BHM-strong	**4%**	73%	78%	71%
5	Independent	57%	57%	61%	53%
	BHM-weak	57%	57%	61%	53%
	BHM-strong	83%	82%	87%	81%

*BHM* Bayesian hierarchical models

Type I error rates are shown in boldface; values not presented in bold indicate power

### Operating characteristics with weak informative prior distribution for $p_0$

We investigated the operating characteristics of the proposed method under the assumption that the prior distribution of $p_0$ was weak informative (Table 7). We assumed that $p_0$ followed a beta distribution with parameters *Beta*(0.05, 0.95) (i.e., the effective sample size (ESS) is one) and a mean of 0.05. Other simulation settings were the same as those described in the Simulation settings section. In the following simulation studies, the values of $c_1$ and $c_2$ were selected to achieve a well-approximated FWER of lower than and nearest to 5% using the proposed numerical approach.

**Table 8:** Well-approximated type I error rates and power with miss-specification of the prior distribution for $p_0$

			Subpopulation *j*			
Scenario	Method	$p_0$	1	2	3	4
1	Independent	Beta(0.1,0.9)	**0%**	**0%**	**0%**	**0%**
		Beta(0.2,0.8)	**0%**	**0%**	**0%**	**0%**
	BHM-weak	Beta(0.1,0.9)	**0%**	**1%**	**1%**	**1%**
		Beta(0.2,0.8)	**0%**	**1%**	**1%**	**1%**
	BHM-strong	Beta(0.1,0.9)	**1%**	**1%**	**1%**	**1%**
		Beta(0.2,0.8)	**1%**	**1%**	**1%**	**1%**
2	Independent	Beta(0.1,0.9)	**0%**	**0%**	**0%**	54%
		Beta(0.2,0.8)	**0%**	**0%**	**0%**	54%
	BHM-weak	Beta(0.1,0.9)	**1%**	**1%**	**1%**	61%
		Beta(0.2,0.8)	**1%**	**1%**	**1%**	62%
	BHM-strong	Beta(0.1,0.9)	**2%**	**3%**	**3%**	65%
		Beta(0.2,0.8)	**2%**	**2%**	**3%**	65%
3	Independent	Beta(0.1,0.9)	**0%**	**0%**	61%	54%
		Beta(0.2,0.8)	**0%**	**0%**	61%	54%
	BHM-weak	Beta(0.1,0.9)	**1%**	**1%**	72%	65%
		Beta(0.2,0.8)	**1%**	**1%**	72%	65%
	BHM-strong	Beta(0.1,0.9)	**5%**	**7%**	83%	76%
		Beta(0.2,0.8)	**5%**	**7%**	83%	76%
4	Independent	Beta(0.1,0.9)	**0%**	57%	61%	53%
		Beta(0.2,0.8)	**0%**	57%	61%	53%
	BHM-weak	Beta(0.1,0.9)	**1%**	70%	76%	68%
		Beta(0.2,0.8)	**1%**	70%	75%	68%
	BHM-strong	Beta(0.1,0.9)	**10%**	85%	91%	84%
		Beta(0.2,0.8)	**10%**	85%	91%	84%
5	Independent	Beta(0.1,0.9)	57%	57%	61%	53%
		Beta(0.2,0.8)	57%	57%	61%	53%
	BHM-weak	Beta(0.1,0.9)	75%	73%	78%	71%
		Beta(0.2,0.8)	74%	73%	78%	71%
	BHM-strong	Beta(0.1,0.9)	91%	90%	95%	90%
		Beta(0.2,0.9)	91%	90%	95%	90%

*BHM* Bayesian hierarchical models

Type I error rates are shown in boldface; values not presented in bold indicate power

Table 7 shows the well-approximated type I error rates and power in scenarios 1-5. We found that the power observed with weak informative prior distribution for $p_0$ was lower than that observed with strong informative prior distribution for $p_0$ in Table 4; however, the power observed with weak informative prior distribution for $p_0$ was still enough when using the BHM-strong method.

### Operating characteristics with miss-specification of prior distribution for $p_0$

We investigated the operating characteristics of the proposed method under the assumption that the prior distribution for $p_0$ was miss-specified (Table 8). We set $p_0$ to fit a beta distribution pattern with the following parameters: (i) *Beta*(0.1, 0.9) with a mean of 0.1, or (ii) *Beta*(0.2, 0.8) with a mean of 0.2; however, the true value of $p_0$ is 0.05. Other simulation settings were the same as those described in the Simulation settings section. In the following simulation studies, the values of $c_1$ and $c_2$ were selected to achieve a well-approximated FWER of lower than and nearest to 5% using the proposed numerical approach.

Table 8 shows the well-approximated type I error rates and power in scenarios 1-5. The values of well-approximated type I error rates and power were very similar with assumptions of *Beta*(0.1, 0.9) and *Beta*(0.2, 0.8). The well-approximated type I error rates and power for the BHM-weak and the independent methods were similar to those under the prior distribution for $p_0$ are correctly specified in Table 4. Otherwise, the well-approximated type I error rates and power for the BHM-strong method were slightly higher than those observed when correctly specifying the prior distribution for $p_0$ in Table 4. However, since the well-approximated FWER of the BHM-strong method was controlled to a value lower than 5%, the proposed method can be used even if the prior distribution for $p_0$ is miss-specified.

## Discussion and conclusion

Explicit control of FWER provides more reliable evidence of efficacy in the final analysis, even in basket trials, and improves decision-making for regulatory drug approvals. Existing Bayesian methods often control the empirical FWER using simulation-based procedures, but the FWER is not controlled when some assumptions are violated. In this study, we provided closed-form equations for the type I and II error rates and FWER in the context of the proposed Bayesian two-stage design and developed a numerical approach for determining the thresholds for controlling the well-approximated FWER to the exact value. To achieve more accurate control of the well-approximated FWER, the proposed method can also be used to adjust the threshold of $c_2$ based on the actual sample size during the final analysis. The simulation studies demonstrated that the proposed design can be used to control the well-approximated FWER below the target value even in situations where the number of enrolled patients differs among subpopulations.

This study showed that the proposed method could weakly control the FWER. However, from a regulatory perspective, strong control of the FWER is necessary, especially in confirmatory basket trials. Therefore, methodological studies of theoretical control of FWER at a target level are required.

In the Bayesian context, controlling the exact FWER is less fundamental when evaluating the therapeutic effect but should be well-calibrated at the planning stage of trials [7–13]. Before beginning the trials, intensive simulation studies are often required to investigate the empirical FWER under various scenarios with respect to the prior distribution, assumed therapeutic effect, sample size, and other factors affecting the therapeutic effect. In contrast to such methods, the proposed method can easily obtain a well-approximated FWER using the proposed numerical approach. Particularly, it is typically necessary to repeatedly calibrate the design parameters in simulation studies to determine the thresholds in most existing basket designs, whereas this is not the case in the proposed method. Although the proposed method focused on controlling the well-approximated FWER, the subpopulation-specific type I error rates and power cannot be controlled at the desired level under a limited sample size. To overcome this issue, it may be better to control the false discovery rate. The extension of the proposed method for controlling the false discovery rate should be further evaluated.

We estimated the posterior probability using the independent and BHM methods in this study; however, other Bayesian dynamic borrowing information models for estimating the posterior probability, such as the extended BHM, the exchangeability-non exchangeability (EXNEX) approach, or Bayesian model averaging can also be used [5–13]. The calculation time of 10,000 simulations for BHM was approximately 24 h with a standard computer (e.g., Intel Core i7 1.1 GHz 1.61 GHz, 16 GB RAM). Therefore, it may take days to finish the calculations for basket trials with a larger number of subpopulations (e.g., $\ge$10 subpopulations) or when using other more computationally complicated Bayesian dynamic borrowing information models. In practice, the accrual number of patients is sometimes unable to reach the pre-defined value; therefore, existing basket designs sometimes cannot ensure the operating characteristics defined before beginning the trial. However, the proposed design enabled adjustment of the cutoff value to control the FWER at the target value based on the results in the final analysis. Additionally, the formulation concept for the type I error rates and the numerical calculation method for controlling the FWER are generalizable. Although the single-arm basket design was the proposed design in this study, we can extend the proposed design to the other types of basket designs such as randomized basket designs.

## Data Availability

Not applicable.

## Abbreviations

BHM: Bayesian hierarchical model
FWER: Family wise error rate
